# Rehabilitation needs of people with brain tumours in Ireland: Protocol for a prospective, mixed methods action research study (“Brain-RESTORE”)

**DOI:** 10.12688/hrbopenres.13786.1

**Published:** 2023-09-25

**Authors:** Ailish Malone, Bernadine O'Donovan, Paul Carroll, Sarah Donnelly, Eloise Cowie, Liam Grogan, Stephen MacNally, Mary O'Sullivan, Jan Sorenson, Eoin J. Tiernan, Rory J. O'Connor, John MacFarlane, Sorcha O'Keeffe, Andrew W. Murphy, Fiona Keegan, Frances Horgan, Kathleen Bennett

**Affiliations:** 1 School of Physiotherapy, Royal College of Surgeons in Ireland, Dublin, Leinster, Ireland; 2 School of Population Health, Royal College of Surgeons in Ireland, Dublin, Leinster, Ireland; 3 National Clinical Program for Rehabilitation Medicine, Health Service Executive, County Dublin, County Dublin, Ireland; 4 Rehabilitation Medicine, National Rehabilitation Hospital, St Vincent’s University Hospital and the Royal Hospital Donnybrook, Dún Laoghaire, County Dublin, Ireland; 5 School of Social Policy, Social Work and Social Justice, University College Dublin, Dublin, Leinster, Ireland; 6 Neuro-oncology, Beaumont Hospital, Dublin, Ireland; 7 Patient and Public Involvement (PPI) Contributor, Kerry, Ireland; 8 Department of Palliative Medicine, St Vincent's University Hospital, Dublin, Ireland; 9 Academic Department of Rehabilitation Medicine, University of Leeds, Leeds, England, UK; 10 Rehabilitation Medicine, Mercy and Cork University Hospitals, Cork, Ireland; 11 Discipline of General Practice, University of Galway, Galway, County Galway, Ireland; 12 Brain Tumour Ireland, Dublin, Ireland

**Keywords:** Brain tumour, rehabilitation, physical function, cognitive function

## Abstract

**Background:** Approximately 480 people annually in Ireland are diagnosed with a primary brain tumour. Brain tumours are a heterogeneous group of conditions, varying in histopathology, location, and progression. A consistent feature is neurological impairment, which can lead to profound effects on physical and cognitive function. There is evidence that people with brain tumours can benefit from rehabilitation, but pathways are poorly described, and no best practice is defined. This leads to significant unmet need. The aim of this study is to understand the rehabilitation needs of people diagnosed with a brain tumour in Ireland, and gain insight to inform policy and practice.

**Methods:** A prospective, mixed methods study with embedded action research will be conducted. Patients (n=122) with a new diagnosis of primary brain tumour, and optionally, a nominated carer or family member, will be recruited through a national neuro-oncology service. Rehabilitation need (Mayo-Portland Adaptability Inventory), quality of life (European Organisation for Research and Treatment of Cancer Quality of Life Questionnaire Brain Cancer Module, EuroQol-5D-5L), healthcare utilisation and, optionally, carer needs (Carer Support Needs Assessment Tool) will be assessed at four, eight and 12 months post diagnosis. An embedded qualitative study will invite 30 patients and carers to a semi-structured interview to explore their lived experience of rehabilitation needs and services following brain tumour diagnosis. Finally, using an Action Research approach, healthcare professionals involved in caring for people with brain tumours will be invited to participate in co-operative inquiry groups, to reflect on emerging aggregate findings and identify actions that could be undertaken while the study is underway.

**Conclusions:** By understanding rehabilitation need, the findings will help healthcare professionals and health service providers understand how to prioritise the supports required and encourage policy makers to adequately resource neurorehabilitation to meet the needs of people with a brain tumour diagnosis.

## Introduction

Every year in Ireland, on average 480 people are diagnosed with primary brain tumours, representing 1.8% of all cancers ^1^. In addition to primary disease, about 9% of people with other cancers ( *e.g.*, breast, lung) will develop secondary brain metastases ^2^. Primary brain tumours are a heterogeneous group of conditions, varying in tumour tissue, location, treatment, complications, individual factors, and progression. Despite the heterogeneity, neurological disability is a consistent feature. Primary brain tumours affect people of all ages but given the different configuration of services for children and adults, this protocol focuses on adults only.

There is a high prevalence of neurological impairment in people with primary brain tumours, creating significant symptom burden ^3^. One controlled trial reported an overall prevalence of limb weakness in 37%, ataxia or limb coordination difficulties in 32%, and sensory-perceptual deficit in 24% of 106 adult survivors of primary glioma ^4^. This symptom profile differs significantly from other cancers and profoundly impacts functional capacity, with up to 47% of people with gliomas in measuring with a Karnofsky Performance Status Score of <70 (“unable to carry on normal activity or do active work”) ^5^. The interaction between these impairments and personal and environmental contextual factors ^6^ leads to participation restrictions including loss of ability to work or drive, relational strain, and risk of poverty, with profound effect on the quality of life of brain tumour survivors.

Rehabilitation is defined as “a set of interventions designed to optimise functioning and reduce disability in individuals with health conditions in interaction with their environment” ^7^. Rehabilitation aims to support people to be as independent as possible in everyday activities and enable participation in meaningful life roles by working with the person to address underlying health conditions and their symptoms, modifying their environment, educating in self-management, and adapting tasks for safety and independence. It is an inherently multidisciplinary, highly person-centred approach. Rehabilitation services are widely established for conditions such as stroke, for which rehabilitation is an essential part of usual care. Brain tumours present different rehabilitation challenges in that they may be progressive and present uncertain futures, but these factors do not preclude potential to benefit ^8^.

The evidence base for brain tumour rehabilitation is not well established ^8, 9^, and mostly focuses on patients with glioma, but nonetheless gives a clear signal that rehabilitation has a place in care of people with brain tumours ^10^. One Cochrane review by Kahn and colleagues in 2015 ^8^ (updated from 2013) assessed the effectiveness of multidisciplinary rehabilitation in people after primary brain tumour treatment, with particular focus on the types of approaches that are effective (settings, intensity) and found that brain tumours can cause significant disability, which may be amenable to multidisciplinary rehabilitation. A more recent systematic review of eight primary studies, including 375 patients with glioma, found that rehabilitation can improve functional prognosis (both motor and cognitive) and quality of life ^10^. A further randomised controlled trial (RCT) of an intensive six-week, thrice-weekly rehabilitation protocol for people with newly diagnosed glioma, compared to a “usual care” control, failed to detect a difference in the primary outcome measure of quality of life, but nonetheless found significantly improved aerobic power and lower and upper limb strength ^5^. That these improvements occurred during active anticancer treatment (chemo- and radiotherapy), a time when a decreased level of functioning might be expected, is particularly notable.

Despite this promising evidence, unfortunately, people with brain tumours often do not get the opportunity to access rehabilitation ^11^. Rehabilitation approaches for brain tumours are unclear in clinical practice guidelines and no current best practice is defined ^5^. The National Institute of Clinical Excellence (NICE) guidelines for brain tumours (primary) and brain metastases in adults, updated in January 2021 ^12^, found limited evidence, though the committee concurred that rehabilitation is likely to be suitable for many people with brain tumours. The NICE recommendations also highlighted that rehabilitation should be considered at every stage of treatment and follow-up. However, other authors have noted that, in practice, the absence of clear pathways and uncertainty about anticipated benefit of rehabilitation leads to barriers to access ^13^. The scale of this problem is difficult to quantify: the proportion of people referred, accepted or declined for rehabilitation is not known ^13^. In Ireland, our team’s experience is that people with a brain tumour make up a small percentage of people referred for neurological rehabilitation and thus their ‘voice’ and presence in the system is relatively small.

## Aims/Objectives

The aim of this study is to understand the rehabilitation needs of people diagnosed with a brain tumour, and gain insight into the pathways towards rehabilitation to inform policy and practice. Specific objectives are:

1. To measure the physical, cognitive and quality of life impacts of a brain tumour on patients and family members and determine how these change over the first year of diagnosis, using quantitative standardised patient-reported outcome measures;
2. To explore patient and family lived experience of rehabilitation need;
3. To measure use of healthcare services in the year following brain tumour diagnosis;
4. To identify potential changes to current practice that could improve patient experiences and outcomes, using an action research approach;
5. To disseminate the findings to key stakeholders, including brain tumour survivors and their families, healthcare professionals and policy makers.

## Methods

### Ethics

Ethical approval has been granted by the Beaumont Hospital Ethics (Medical Research) Committee (REC ref: 23/21).

### Study design

This is a prospective longitudinal mixed methods study with embedded action research. The study will recruit people with brain tumour at time of diagnosis, assess the symptom burden, level of disability, and rehabilitation needs and measure how these change over a period of one year after diagnosis.

The data management plan, participant information sheet, consent form and interview topic guide can be found as *Extended data* ^14^.

The next sections will describe the methods pertaining to patient and carer participants in the quantitative and qualitative data collection. In terms of sex and/or gender analysis, men are more likely than women to develop brain tumours. Participants will be purposively sampled according to gender and all qualitative data will be analysed by gender. Our research materials will be piloted to assess the appropriateness of the language and any differences in interpretation between genders. Gender-neutral language will be used to avoid gender bias. Efforts will be made to ensure balanced representation of people with brain tumour and their family members, by both genders in the PPI advisory group. Gender balance will be achieved in dissemination activities by reporting and disseminating findings in a gender-sensitive form, publishing results that have been gender-differentiated, employing gender-neutral language and involving gender-related institutions among the target audiences. There are no biological (sex) considerations for this research.

### Patient and carer participants

Potential patient participants will be identified through the multi-disciplinary meeting (MDM) of the neuro-oncology service at Beaumont Hospital, Dublin. Beaumont Hospital is a National Cancer Care Programme (NCCP) Designated Cancer Care Centre for neuro-oncology. Approximately 500 people with new incidence of brain tumour (primary and secondary, all grades) are referred to the service annually.

This research strives to account for and measure all potential rehabilitation needs of people with brain tumours who may experience sensorimotor or cognitive deficits. Eligibility criteria therefore include several tumour types. The criteria recognise that although histology and grade predict medical management, rehabilitation needs will vary within, as well as between, tumour types.

***Inclusion criteria***

1. New diagnosis, confirmed by a consultant in the neuro-oncology service, Beaumont Hospital (Neurocent Directorate), of one of the following primary brain tumour types from the WHO 2021 Classification of Central Nervous System tumours:
  a. Glioma, glioneuronal and neuronal tumour, ependymoma,
  b. Cranial nerve tumour,
  c. Meningioma;
2. Tumour located in the cerebral hemispheres or posterior fossa region;
3. Age minimum 18 years;
4. Capacity to consent, or, for those with cognitive impairment, to consent with a decision supporter in accordance with the Assisted Decision-Making (Capacity) Act 2015;
5. Medically well enough to participate, as determined by the neuro-oncology multidisciplinary team.

***Exclusion criteria***

1. Diagnosis of the following brain tumour types (WHO, 2021):
  a. Choroid plexus tumours,
  b. Embryonal tumours,
  c. Pineal tumours,
  d. Mesenchymal, non-meningothelial tumours,
  e. Melanocytic tumours,
  f. Haematolymphoid tumours,
  g. Germ cell tumours,
  h. Neuro-endocrine tumours of the sellar region and craniopharyngiomas,
  i. Metastases,
  j. Genetic tumour syndromes involving the CNS;
2. Tumour located in the sellar region, skull base or ventricular system;
3. Cognitive deficit of such severity that it is not feasible to assess outcome measures, even with adaptation or involvement of family members;
4. Predicted survival less than three months from time of diagnosis;
5. Co-existing neurological disorder that could confound assessment of rehabilitation needs.

### Recruitment

Recruitment will take place over a 12-month period. People with brain tumours who meet the inclusion criteria will be identified by a neuro-oncology Clinical Nurse Specialist (CNS). A member of the neuro-oncology CNS team consults with all patients as part of usual care during their inpatient stay. Once the diagnosis of brain tumour type is confirmed at the neuro-oncology multidisciplinary meeting and the clinical plan determined, the CNS will seek agreement of the neuro-oncology lead consultant (co-author SMN, or nominee) to share information about the study.

The timing of sharing information will be carefully considered for each individual patient. People with brain tumours face an overwhelming amount of information in the early days and weeks after diagnosis, particularly during an inpatient admission. For this reason, information about the study will be shared at least four weeks after histopathology diagnosis, in the course of routine outpatient follow-up.

**Figure 1.**
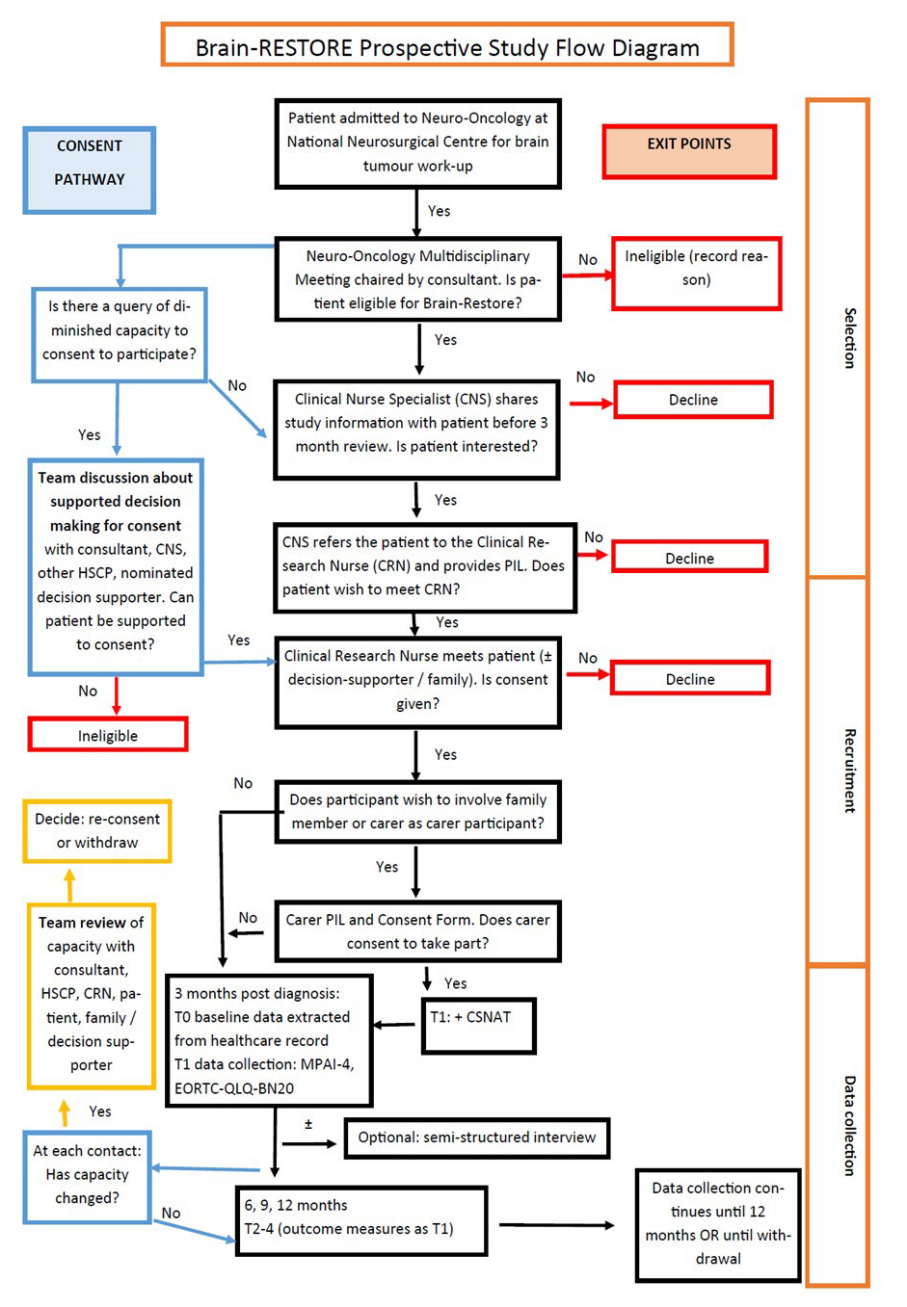
Recruitment Flow diagram for WP4A and 4B (patient and carer participants) including procedure for consent. Presentation of process for selection and recruitment of participants with brain tumour with details of consent pathway.

The CNS will share information about the study initially *via* verbal communication and / or a one-page flyer, either on a routine phone follow-up or an outpatient appointment. If the patient expresses interest in taking part, the CNS will share the full Participant Information Leaflet (PIL) and ask their permission to refer them to the Clinical Research Nurse (CRN). The CRN will then arrange to meet with the patient to discuss the study, explain the procedures and answer any questions. Before inviting consent, the CRN will confirm that the patient has read and understood the PIL, in the presence of a family member or nominated carer if decision-making and participation is being supported by this person. The participant will then be invited to sign explicit and informed consent. If the person with a brain tumour wishes to nominate a carer or family member to participate, the carer or family member will be invited to complete a Carer Consent Form. Figure 1 shows the process for selection and recruitment.

***Considerations for recruiting participants with cognitive impairment***

People with brain tumours may have a significant symptom and treatment burden, including the possibility of fluctuating cognitive impairment. Cognitive impairment, whether temporary, fluctuating or established, forms a significant part of the symptom burden for people with brain tumours and their families so it is important that they are included, where possible, in this research. Otherwise, the findings will be biased towards people who have no cognitive impairment and will not be representative of the population.

Participants will therefore be selected and recruited in consultation with the treating Consultant. Decisions pertaining to capacity to consent will be made at a clinical level, following the HSE’s National Consent Policy. The team will be guided by best practice, as underpinned by the Assisted Decision-Making Act, 2015 (ADMA) in ensuring that accommodations and supports are in place to maximise the capacity of all potential participants to provide informed consent to participate in the study. It is recognised that a person’s capacity to consent is assumed unless proven otherwise. It is also recognised that, particularly for a brain tumour undergoing active treatment, cognitive deficits can be transient or fluctuate, and respond rapidly to interventions such as corticosteroid treatment. Therefore, we aim to give every patient with a brain tumour the opportunity to participate, in consideration of the balance of risks and benefits and in accordance with the family’s understanding of the person’s will and preferences. Where a potential participant with cognitive impairment indicates a wish to take part, a family member or carer will be invited to be a decision supporter in line with the decision-making support structures under ADMA legislation. If there is sufficient trigger to query capacity to consent, the matter will be discussed between the consultant and one of the healthcare professionals designated in ADMA. Procedures for recruitment and consent are shown in Figure 1.

The outcome measures proposed for baseline and follow-up assessments are designed to be completed either independently or with involvement of family. In the event that data collection of all outcome measures is curtailed or affected by the presence of cognitive impairment, this will be noted as an outcome in itself and will be considered in statistical analysis.

***Sample size***

The sample size calculation was based on the Mayo-Portland Adaptability Inventory (MPAI-4). Other studies have shown meaningful improvements in this outcome measure, based on a similar cohort of acquired brain injury patients in rehabilitation, of a 5 T-score point change (assuming a SD=10, equivalent to an 0.5 SD change) as a minimally clinically important difference for interventions ^15^. Assuming the change over 12-months is of similar magnitude in the proposed study, then the sample size required is n=61. This assumes the correlation between baseline and follow-up measure is r=0.5, 90% power and 5% level of significance. If we assume a conservative 50% dropout over the follow-up, then n=122 will be recruited.

### Prospective study quantitative data collection

***Baseline assessment***

Baseline data at diagnosis (T0) will be recorded at time of enrolment to the study. These data will be obtained from the healthcare record at admission for surgery.

1. Brain tumour grade (WHO 1-4) and histology (descriptive, cell type);
2. Age at diagnosis;
3. Past Medical History or co-existing medical conditions, coded using the World Health Organisation International Classification of Diseases 11th Revision (ICD-11);
4. Social history (living alone or with family member), occupation (descriptive);
5. Deficits caused by brain tumour at time of presenting to the health services (descriptive);
6. Karnofsky Performance Status (KPS) scale and Eastern Cooperative Oncology Group (ECOG) Performance Status ^16^. The KPS and ECOG involve rating the patient’s functional status on an ordinal scale based on the findings of assessment and do not impose additional assessment burden on the patient.

***Outcome measures***

Follow-up assessments will be conducted four months (T1), eight months (T2) and 12 months (T3) following diagnosis. Data collection will take place in person at a scheduled review clinic in Beaumont Hospital. Most people with brain tumours will attend several follow-up appointments so the timing of research data collection will be aligned with these existing clinical appointments, to avoid an additional burden on the participants and their families. Where there is no clinical appointment, or if it is not possible for the participant to travel, data collection will be conducted remotely *via* videoconference or telephone, at the participant and family member’s preference.

Physical and cognitive disability will be assessed using the Mayo-Portland Adaptability Index (MPAI-4) ^17^, a widely used measure of limitations resulting from acquired brain injury. It is a 30-item scale giving a total score reflecting overall disability, and three subscale scores for Ability (including mobility, cognition, communication), Adjustment (including pain and fatigue) and Participation (including independent living, employment, and social contact). To our knowledge, there is no measure of neurological disability that has been specifically developed and validated for people with brain tumours. The MPAI has been previously reported for complex neurological disability, particularly Traumatic Brain Injury, and its minimal clinically important difference (MCID) was established in a large mixed population that included some people with brain tumours ^15^. It demonstrates satisfactory internal consistency, construct validity, concurrent and predictive validity for the full measure and its three subscale scores ^18^. It can be completed by a healthcare professional, patient, or significant other, or by a team of clinicians, giving flexibility in consideration of the potential challenges of cognitive impairment.

The European Organisation for Research and Treatment of Cancer Quality of Life Questionnaire Brain Cancer Module (EORTC QLQ-BN20) will be used to evaluate patient-reported health-related quality of life (HRQoL) and symptom burden ^19^. The EORTC-BN20 questionnaire contains 20 items of which 13 cumulate into 4 multi-item scales representing: future uncertainty, visual disorder, motor dysfunction, communication deficit; and seven are single items (headaches, seizures, drowsiness, hair loss, itchy skin, weakness of legs and bladder control). It demonstrates adequate internal consistency, responsiveness and validity ^19^. Additionally, to enable comparison with other conditions and populations, the EuroQol-5D-5L (EQ-5D) will measure perceived health status and HRQoL ^20^.

In the event that the participant nominates a carer or family member to participate, and the carer consents to taking part, then the perspective of carers and family will be sought using the Carer Support Needs Assessment Tool (CSNAT), a validated instrument designed to systematically identify and address caregiver needs ^21^. It is a carer-led, healthcare professional-facilitated 14-item tool, with each item representing a core family carer support domain. The CSNAT has been previously used to assess support needs of family caregivers of patients with brain tumours in Australia ^22^. In this cross-sectional study of 29 caregivers, the CSNAT was found to be useful and practical in measuring the challenging caregiver experience with brain tumours and was recommended for use in future prospective longitudinal studies that could determine evolving caregiver needs at different disease stages.

Healthcare utilisation will be recorded by self-report of visits to different healthcare providers (including General Practitioners, Emergency Department, Outpatients, and others) that occurred in the previous three months, using a standardised checklist. Referrals to rehabilitation services and palliative care will be noted.

***Participant retention***

The course of a brain tumour varies. A participant’s symptom and treatment burden may change over time and not all participants will be able to complete. In addition to voluntary discontinuation or loss to follow-up, anticipated endpoints include:

- Withdrawal due to becoming too medically unwell to continue;
- Death within 12 months of diagnosis.

***Statistical analysis***

Descriptive statistics will be presented including means (standard deviations), medians (inter-quartile range) or frequencies (proportions). The primary analyses will examine change in measures (MPAI-4, EORTC QLQ-BN20 and CSNAT) over time from baseline to final follow-up using linear mixed models or generalised linear mixed models (for longitudinal analysis). Mixed modelling will be used to identify associations between changes over time and factors such as the type or grade of tumour, patient characteristics such as age, previous medical history and surgery and treatments received. Total utilisation of rehabilitation services (specialist or through local primary care teams) will be described and associations with tumour, clinical and patient characteristics examined using generalised linear models for count data. Survival at the end of the one year will be examined using Kaplan-Meier plots. Statistical analysis will be conducted using SAS (v9.4) or Stata (version 17.0). Significance at p<0.05 will be assumed.

### Qualitative interviews with patients and family

***Participants***

In total, 30 of the 122 participants in the prospective study and their carers / family members will be invited to participate in semi-structured interviews to explore their lived experience of the impact of a brain tumour on physical and cognitive function, and their perceptions of rehabilitation need. Semi-structured interviews will take place between six and 12 months after diagnosis. At the time of enrolment to the prospective study, participants will be asked to indicate willingness to take part in the semi-structured interviews. Recruitment will continue until 30 participants enrol or until saturation is reached; that is, when no new information emerges ^23^.

***Data collection***

Interviews will be conducted by an experienced Post-Doctoral Researcher. We will provide participants with the option to conduct interviews over the telephone or *via* an online platform of their choice. Interviews will be guided by topic guides. Topic guides will be developed collaboratively by the project management team, PPI group, researchers and people diagnosed with a brain tumour and their families. The topic guides will be piloted with at least two patients prior to use. Interviews will be audio-recorded and transcribed verbatim for analysis.

***Analysis***

Interview transcripts will be analysed using six staged Braun and Clarke reflexive thematic analysis ^24^: 1) Familiarising with the data; 2) Generating initial codes; 3) Searching for themes; 4) Reviewing themes; 5) Defining and naming themes; and 6) Producing the report. Data will be analysed inductively, allowing themes to arise from the data using a bottom-up approach. Finally, a deductive approach will be completed to provide a comprehensive understanding of the data studied. The post-doc, a co-applicant and the Lead Applicants will discuss emerging themes collaboratively to enhance the depth of interpretation. Strategies to enhance trustworthiness of the findings and reflexivity, will be used. Data will be managed using NVivo software.

***Distress protocol***

During both quantitative and qualitative data collection, it is possible that participants could become distressed. The researcher will observe for any potential indications of distress. Brain tumours can lead to changes in emotional regulation ^25^ so the interpretation of distress will be considered in the context of what is normal for the participant. If there is an indication that the interview itself is causing distress, the researcher will stop the recording and will talk to the participant about their distress. The participant will be offered to take a break, end the data collection or interview, or continue talking. The decision of the participant is final.

Scenario 1: If the participant decides to take a break and continue with the interview, it will be confirmed if they are comfortable to continue. The participant will be reassured that they can stop the interview or withdraw at any time. The researcher will encourage the participant to seek support from the neuro-oncology CNS or their GP, and will signpost to other psychological support services such as Pieta and Samaritans, or general supports such as Brain Tumour Ireland, Family Carers Ireland and Irish Cancer Society.

Scenario 2: If the participant does not want to continue, the interviewer will remain with them and give them an opportunity to de-brief to ensure the participant is not visibly distressed when leaving the interview. The researcher will encourage the participant to seek support from the professionals and organisations above.

### Participatory Action Research

A fundamental motivation for this study is to generate findings that may facilitate real-world improvement in rehabilitation service provision, for people affected by brain tumours in Ireland. Action research is particularly suited to identifying problems in clinical practice and helping develop potential solutions in order to improve practice and outcomes for the patients and families that we work with. Research in this area involves witnessing of need by the researcher and this witnessing asks of the researcher, how will they respond to their findings as they conduct their study? Given the focus of the study, we see it as ethically problematic to conduct a study whose design gathers data over 2–3 years and simply compiles an academic report at the end of the study. For some of the potential patient participants 2–3 years may be all or a major part of their remaining life. Time is therefore of profound importance. Noting this we will be embedding an Action research ethos to the project with the aim of converging research findings and clinical practice, to foster better practice across interprofessional boundaries and between different healthcare settings.

***Action Research Approach***

An action research approach has been chosen to describe, evaluate and offer a mechanism for the development of service delivery as it is inherently practical, change orientated, cyclical and participatory in nature. Action Research can be any systematic enquiry, either large or small, conducted by professionals and focusing on some aspects of their practice in order to find out more about it and eventually to act in ways that they see as better or more effective. Research is rooted in participation and therefore done with rather than on participants who often become co-researchers and is an ongoing organisational learning process that emphasises co-learning, participation and organisational transformation. A central tenet within Action research is asking ‘how might we change things at the same time as studying them’ (McTaggart, 1997, p.26) ^26^. Action research therefore involves a cycle/cycles comprising of inquiry, intervention, and evaluation in contrast to a more traditional research approach which could be summarised as inquiry, data analysis and dissemination of results.

***Participants in Action Research: Healthcare professionals as co-participants and co-researchers***

An action research approach will be embedded through the use of a co-operative inquiry group process. With a cooperative inquiry approach, each group member becomes both a co-researcher and a co-subject in the inquiry ^27, 28^. The key focus in understanding cooperative inquiry is firstly, how each person is both a co-subject in the experience phases *via* their individual experiences being the subject of the inquiry and secondly, a co-researcher in the reflection phases by participating in shared inquiry through sharing experiences, questioning and drawing out individual and shared learning ^29^.

Biannual cooperative inquiry groups will be convened to reflect on salient findings as they emerge over the two-year timeframe of the study. Members of the research team, clinical professionals working with people with brain tumours, and PPI panellists will be invited to participate in the co-operative inquiry group by the post-doctoral researcher, who will provide an Action Research Participant Information Leaflet. If they wish to take part, Informed Consent will be sought at the start of the co-operative inquiry group. The co-operative inquiry group process will facilitate the identification of the interests of those who are meant to be served by the changes to practice or service delivery ^30^ *i.e.*, the brain tumour population. It will also provide the opportunity to explore and respond to presenting problems in relation to rehabilitation for this population, offering a mechanism for understanding the current problems, then acting and reflecting on this emergent knowledge. It will facilitate communication of these findings to clinicians involved in service provision and elicit the views of clinicians and service users with regard to their experience of service as it is delivered and modified, through systematic ongoing reflective practice on the part of the researchers.

***Outcomes of Action Research***

The adoption of an Action Research approach enables the team to reflect on, and respond to, presenting problems and emergent knowledge identified through standardised prospective re-assessment over the year following brain tumour diagnosis. We anticipate this study may yield findings, as it progresses, which if acted on, could bring about improvement in rehabilitation service provision, outcomes and patient experiences. We will record the following qualitative and quantitative outcomes of this approach:

1. The gaps in service identified through prospective follow-up. A gap will be defined as the need for review by a healthcare professional or service that had not been already actioned through routine clinical care.
2. The number of onward referrals made by the Clinical Research Nurse in response to these identified gaps, and the response to these referrals.
3. The number of subsequent patient encounters resulting from these onward referrals.

The co-operative inquiry process will utilise cycles of reflecting, planning and action through a relational, reflexive process of mutual engagement to reflect on emergent knowledge generated from these outcomes and facilitate discussion about what changes to practice should be made. In this way, qualitative and quantitative findings will be used to inform changes to current processes and practice culminating in the development of best practice guidelines for the rehabilitation of brain tumour patients.

***Clinical governance of action research***

In an Action Research Cooperative Inquiry process, individual patient participants would not be routinely discussed and the focus would be more on emerging general issues. Nonetheless, following the general discussion and in consideration of the time sensitive nature of rehabilitation needs in people with brain tumours, the actions arising from the action research and cooperative inquiry process may include individual interventions such as onward referral, where this had not already been done and where it could be unethical not to do so. The Clinical Research Nurse will inform the participant’s treating consultant about any actions taken, or that need to be taken.

## Public and patient involvement

This proposal has been developed with public and patient involvement from two representatives, who are co-authors and were a co-applicant and collaborator, respectively, on the application for funding. Prior to study commencement, a public and patient involvement (PPI) panel will be convened, to be made up of four to six people with lived experience of rehabilitation needs arising from a brain tumour journey. Cognisant of the significant burden of living with a brain tumour, the team will create flexible arrangements for PPI panellists to input at a time that suits them. Tasks to be assigned to the PPI panel may include review of the study materials (PIL and consent forms, data collection procedures and semi-structured interview schedule), input to the Action Research co-operative inquiry groups, advice on the design of the study webpage and guidance for public dissemination.

## Data management

A Data Management Plan has been developed. All data will be stored securely on a shared drive with restricted access, with multifactor authentication in place for additional protection.

Data will be pseudonymised. Following completion of data collection and data checking / validation, all data will be irrevocably anonymised.

Audio recordings from qualitative semi-structured interviews will be uploaded to SharePoint and transcribed. Following checking and validation of transcription, the audio file will be deleted. Identifying details will be redacted from the transcript.

## Reporting of results

The prospective study will be reported according to the Strengthening the Reporting of Observational Studies in Epidemiology (STROBE) guidelines ^31^. Semi-structured interviews will be reported according to the COnsolidated criteria for REporting Qualitative research (COREQ) guidelines ^32^.

## Dissemination

The results of this study will be shared within the scientific community through peer reviewed publications and national and international conferences. Findings from Action Research will be summarised into recommendations for practice. Public dissemination will take the form of infographics and videos designed for sharing on social media and on the study’s website, https://brainrestore.eu. Opportunities for public and patient dissemination will be explored through the study’s charity collaborator, Brain Tumour Ireland.

## Study status

Recruitment and data collection is scheduled to commence in October 2023.

## Discussion

Brain tumour rehabilitation is complex and challenging, and in light of recent initiatives as outlined in the United States National Coalition for Cancer Survivorship (NCCS) and Irish National Cancer Care Programme (INCCP), which aims to produce evidence‐based guidelines and implement survivorship care plans, there is a need to address the long‐term requirements of cancer survivors. Advances in medical care and increased life expectancy among people with disabilities mean that ongoing health and well‐being becomes increasingly important and requires longer‐term planning. From a rehabilitation perspective, the challenge is not just about helping the brain tumour survivor to overcome the symptoms and improving their performance status; it is also about helping them stay independent in their community in light of changes associated with tumour progression or recurrence, as well as ageing, and helping their families to overcome the additional demands and stress. A better understanding of the optimal structure, function, timing and content of multidisciplinary rehabilitation along the recovery trajectory would guide improvement of service provision from an organisational and economic perspective.

The proposed research will impact a range of stakeholders including those diagnosed with a brain tumour, their families/carers, healthcare professionals, policy and decision makers and academic researchers. The experience of survivors and carers should help to prioritise the supports required and encourage healthcare providers and policy makers to adequately resource neuro-rehabilitation for those with a brain tumour diagnosis. This should significantly improve outcomes for patients. For healthcare providers, the proposed research will provide evidence on the most effective interventions, and the needs assessment for patients (and carers) using validated tools in practice. For policy and decision makers the impact will be to highlight the deficits in rehabilitation service provision but also provide exemplars of best practice that need to be scaled up for population coverage. Finally, there is limited research on the unmet rehabilitation needs of those diagnosed with a brain tumour in Ireland. The proposed research will contribute to the wider literature and provide data that is lacking at present. Others have identified, in those with acquired brain injury in Ireland, that the key challenges in neuro-rehabilitation include the absence of services across the ‘pathway’, the under-resourcing of specialist rehabilitation services, the impact on the lives of people with poor or no access to services, and the lack of good data on this population, all of relevance to the proposed research.

## Data Availability

No data are associated with this article. Figshare: Rehabilitation needs of people with brain tumours in Ireland: "Brain-Restore". https://doi.org/10.6084/m9.figshare.24175476.v1 ^14^. This project contains the following extended data: Data Management plan Participant information sheet Consent form Interview Topic Guide Data are available under the terms of the Creative Commons Attribution 4.0 International license (CC-BY 4.0).
