# Supplementary material for: Rehabilitation needs of people with brain tumours in Ireland: Protocol for a prospective, mixed methods action research study (“Brain-RESTORE”)
Source: HRB Open Res. 2023 Sep 25;6:58. [Version 1] doi: 10.12688/hrbopenres.13786.1 (PMC11255547; doi:10.12688/hrbopenres.13786.1)
Supplement: Peer review history

# Reviewer report: Reviewer response for version 1

**DOI:** 10.21956/hrbopenres.15083.r39174

**Published:** 2024-08-07

**Authors:** Jennie Taylor

**Affiliations:** 1 University of California, San Francisco, California, USA

Malone et al present their study design for their “Brain-RESTORE” protocol, a prospective, mixed method approach to understanding and addressing rehabilitation in patients with primary brain tumors. They outline the scope of the problem and its potential impact; the study population; methods; and analysis plan. Given that this project has already received funding and anticipate starting accrual in 10/2023,  this manuscript as written seems to address their intent to outline their protocol and is acceptable for indexing as written.

Is the study design appropriate for the research question?

Yes

Is the rationale for, and objectives of, the study clearly described?

Yes

Are sufficient details of the methods provided to allow replication by others?

Yes

Are the datasets clearly presented in a useable and accessible format?

Not applicable

Reviewer Expertise:

NA

I confirm that I have read this submission and believe that I have an appropriate level of expertise to confirm that it is of an acceptable scientific standard.

# Reviewer report: Reviewer response for version 1

**DOI:** 10.21956/hrbopenres.15083.r39953

**Published:** 2024-07-17

**Authors:** Georgia K B Halkett

**Affiliations:** 1 Curtin University, Perth, Western Australia, Australia

Thank you for the opportunity to review this article. This is an important study and the protocol is presented well. Here is some feedback for consideration. 

1. The number of people with primary brain tumours in Ireland is quoted as 480 in the abstract and background. However, in the methods the authors state that approximately 500 people are referred to the service annually. This needs to be clarified to highlight how many might have secondary tumours and hence ineligible for the study.
2. More information about the different types of primary brain tumours could be included in the background.
3. Good background of previous studies in this area relating to rehabilitation. It would also be good to include studies on exercise during treatment for brain tumours.
4. Aim 5 is not being measured -it could be removed and in the discussion/conclusion authors could highlight this is the plan.
5. Great to provide detailed recruitment plan
6. What is the timeline for recruiting 122 participants? This may be difficult to achieve in one year.
7. Appropriate instruments have been selected with carer involvement included.
8. Great to see qualitative interviews also included with plan for thematic analysis
9. Action research is proposed. How will challenges/problems be documented? More detail could be provided around this.
10. Well written discussion. 

Is the study design appropriate for the research question?

Yes

Is the rationale for, and objectives of, the study clearly described?

Yes

Are sufficient details of the methods provided to allow replication by others?

Yes

Are the datasets clearly presented in a useable and accessible format?

Not applicable

Reviewer Expertise:

radiation therapy, qualitative and quantitative research, research with patients with brain cancer and carers,

I confirm that I have read this submission and believe that I have an appropriate level of expertise to confirm that it is of an acceptable scientific standard.

# Reviewer report: Reviewer response for version 1

**DOI:** 10.21956/hrbopenres.15083.r37165

**Published:** 2024-05-11

**Authors:** Ingrid Tonning Olsson

**Affiliations:** 1 Lund University, Lund, Sweden

This is a well-designed study addressing an important question. Aims are to assess rehabilitation needs in adults with brain tumors in Ireland, and to gain insight to inform policy and practice. Strengths of the study include the inclusion of care givers/family members as informants, the inclusion of patients with severe disabilities, and a very well-outlined and structured mixed methods design with embedded action research. This research is also important, considering that the researchers address the, often overlooked, needs of a vulnerable group of patients. Other strengths of the proposed study are that it was and will be developed with public and patient involvement, and that the authors have a plan for disseminating results not only in scientific settings but also in clinical guidelines.

 I have some minor remarks:

 No motivation is offered for inclusion criteria or for the rationale for the “Neuro-Oncology Multidisciplinary Meeting” to consider a patient eligible. Also, inclusion and exclusion criteria might be simplified; for example, survivors never included do not need to be excluded (inclusion criteria: primary brain tumor, exclusion: metastases). This might not be important since the aim is not to evaluate if the rehabilitation program is efficient. If the authors, in the future, might want to publish an evaluation of the effectiveness of the rehabilitation program offered, it is important to show that not only patients deemed to do well in the program were selected, or to provide a clear rationale to why patients were selected in the first place.

 For clarity I would avoid using CNS as an abbreviation for Clinical Nurse Specialist, since it is most often used as an abbreviation for central nervous system.

 These are minor remarks and I believe the authors have done an excellent job in designing a comprehensive and well-thought-out study. I am looking forward to reading about the results.

Is the study design appropriate for the research question?

Yes

Is the rationale for, and objectives of, the study clearly described?

Yes

Are sufficient details of the methods provided to allow replication by others?

Yes

Are the datasets clearly presented in a useable and accessible format?

Yes

Reviewer Expertise:

Neurocognitive late complications in survivors of childhood cancer

I confirm that I have read this submission and believe that I have an appropriate level of expertise to confirm that it is of an acceptable scientific standard.
